# Bioactive Molecules in Soil Ecosystems: Masters of the Underground

**DOI:** 10.3390/ijms14058841

**Published:** 2013-04-24

**Authors:** Xuliang Zhuang, Jie Gao, Anzhou Ma, Shenglei Fu, Guoqiang Zhuang

**Affiliations:** 1 Research Center for Eco-Environmental Sciences, Chinese Academy of Sciences, Beijing 100085, China; E-Mails: giji0812@126.com (J.G.); azma@rcees.ac.cn (A.M.); gqzhuang@rcees.ac.cn (G.Z.); 2 Key Laboratory of Vegetation Restoration and Management of Degraded Ecosystems, South China Botanical Garden, Chinese Academy of Sciences, Guangzhou 510650, China; E-Mail: sfu@scbg.ac.cn

**Keywords:** below-ground ecosystem, rhizosphere, bioactive compounds, quorum sensing

## Abstract

Complex biological and ecological processes occur in the rhizosphere through ecosystem-level interactions between roots, microorganisms and soil fauna. Over the past decade, studies of the rhizosphere have revealed that when roots, microorganisms and soil fauna physically contact one another, bioactive molecular exchanges often mediate these interactions as intercellular signal, which prepare the partners for successful interactions. Despite the importance of bioactive molecules in sustainable agriculture, little is known of their numerous functions, and improving plant health and productivity by altering ecological processes remains difficult. In this review, we describe the major bioactive molecules present in below-ground ecosystems (*i.e.*, flavonoids, exopolysaccharides, antibiotics and quorum-sensing signals), and we discuss how these molecules affect microbial communities, nutrient availability and plant defense responses.

## 1. Introduction

Amazingly complex interactions exist within the unseen below-ground environment, including root-root, root-insect, and root-microbe interactions, which can have both positive and negative outcome [1]. These sophisticated processes, which include species host-microbe interactions (e.g., mutualistic or pathogenic relationships), metabolism (e.g., root exudation and parasitic plants), energy transfer (e.g., electric potentials and resource partitioning), and information exchange (e.g., protective biofilms and quorum sensing), play pivotal roles in terrestrial ecosystems [1,2]. Recent research has focused on these complex responses in the below-ground ecosystem for the inspiration of possible solutions to influence plants with a better yield [3].

A wide range of bioactive molecules, often through complex mechanisms, are the main effectors of these associations and modulations. These molecules, in dealing with below-ground ecological system associations, are generated as secondary metabolites by organisms such as bacteria, fungi, lichens, invertebrates, plants, and (most likely) mammals. They are characterized by their capacity to actively modulate biological processes in the soil ecosystem, for example, by stimulating beneficial properties or by interfering with signaling pathways used to interact within or between species [4]. These bioactive molecules in the below-ground ecosystem are derived from multiple types of biosynthesis and provide cell-signaling networks to control the individual physiological process.

Plants depend on microbial bioactive compounds, some of which have functions within the below-ground ecosystem besides the coordination of microbial behaviors. And also, plant roots continuously release a multitude of organic compounds into the rhizosphere, with the intent of recruiting beneficial microorganisms and fending off pathogens, which provide growth advantages and disadvantages to plants, respectively [5]. Environmental factor, such as light intensity, is important to rhizosphere community composition [6]. However, root exudates have been implicated as determinants that can exert stimulatory and inhibitory influences on the community structure and composition of microbes within the rhizosphere, and they influence resource competition, nutrient availability, chemical interference, and parasitism between plants [1,6–8].

Much research is currently focused on the significance of information interactions in below-ground ecosystem. Plant or microbial cells produce signals that go through several phases, including (1) synthesis; (2) release and transmission; and (3) response and feedback acquirement [9]. During the information-transfer process, the expression levels of many different genes are modulated, which might differentially affect the behavior of the plant or the microbial community. Quorum sensing (QS) is an important communication system used during symbiosis, defense, and other interactions between plants and microorganisms, and it appears that this cross-talk system between microorganisms provides information in complex unseen networks.

Bacteria, by sensing the density of their own or other species within a community, can alter their behavior through the activation of gene expression by secreting autoinducing signaling molecules [10]. This process, termed quorum sensing, is a form of cell-cell communication used by bacteria that is mediated by *N*-acyl homoserine lactones (AHLs) as well as other molecules, including *p*-coumarate [11], quinolone [12], and 3OH palmitic acid methyl ester (3OH PAME) [13] in several gram-negative bacteria and oligopeptides in gram-positive bacteria [14]. Moreover, the autoinducer AI-2, which is a furanosyl borate diester produced by *Vibrio harveyi*, is one of several signals that allow bacteria to communicate between species. Diverse biological functions, such as biofilm formation [15], symbiosis [16], virulence [17,18], swarming behavior [19], bioluminescence, and antibiotic production [14], are subject to QS regulation.

**Figure 1:**
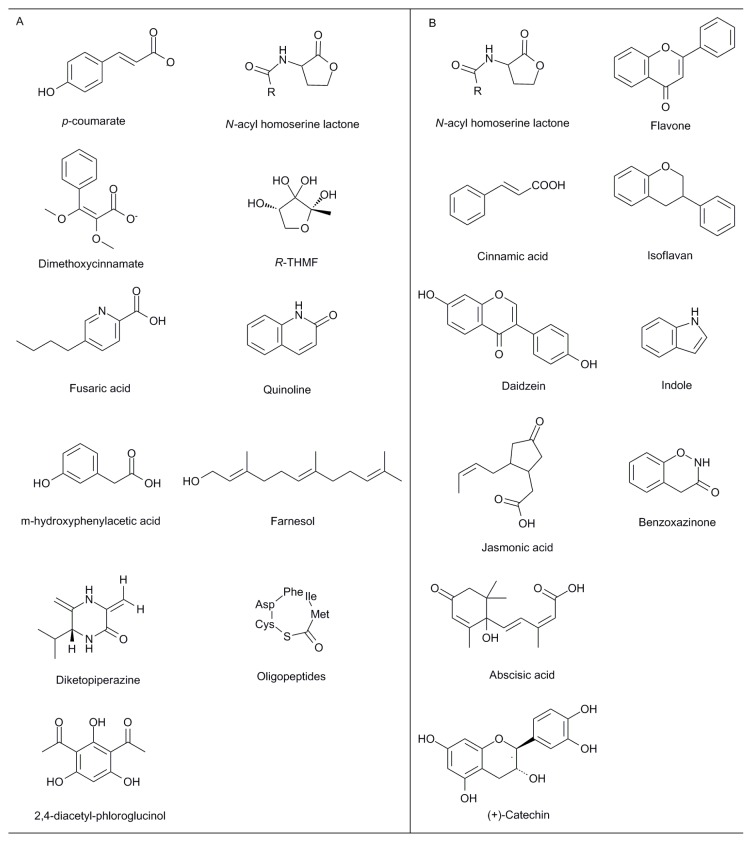
Some basic structures of bio-molecules act as signals in rhizosphere [1,16,20–22]. (**A**) Molecules produced by microbes; (**B**) Examples of root exudates.

Signaling molecules involved in QS are strongly associated with stimulating biological activities and triggering a range of signal transduction cascades during root-microbe interaction processes. This signal-mediated mechanism indirectly controls plant-microbe communication. However, bioactive molecules produced by microbes or as secondary metabolites of roots and other mediators, can also be perceived as broad-spectrum signals, which have both direct and indirect information processing within the rhizosphere (Figure 1). These bioactive molecules include primary factors with (presumably) immediately beneficial or adverse influences on plant and microbial growth and survival (summarized in Table 1). Furthermore, the induction-response mechanism is dominated by an enormous range of low-molecular-weight compounds spread throughout the rhizosphere, which indirectly control plant-microbe communication (Table 2).

The “Underground Revolution” has focused the attention of scientists on how below-ground ecosystems can influence and increase crop yield [3], and indeed, a large number of the molecules produced by living organisms can have major influences on rhizosphere microbial communities and nutrient availability for acquisition by plants [1,57–59]. Therefore, it is crucial that we focus more attention on bioactive molecules in below-ground ecosystems. This review will concentrate on these unseen ecosystems and will describe the interactions between microorganisms, plant roots, and the rhizosphere soil. Specific attention is paid to the biochemical compounds that are secreted into the soil.

## 2. Essential and Regulatory Roles of “Bio-Signals”

### 2.1. Trophic Interactions and C/N/P

Plants are the principal parts of the terrestrial ecosystem, although carbon sequestration, nutrient cycling and productivity are highly reliant on soil microorganisms. Roots have evolved a range of techniques for increasing the availability of essential energy and nutrients, including changes in the growth and development of the root system. However, in some respects, the contributions of rhizospheric microorganisms are even more important for modulating nutrient supply within soil. This type of interaction between plants and microbes sustains the ecological function of below-ground ecosystem.

#### 2.1.1. Nitrogen Fixation

Biological nitrogen fixation is mainly driven by soil bacteria, called rhizobia, which forms symbiotic associations with roots. Nitrogen fixation by rhizobia is one of the best-studied examples of root-microbe interactions [60]. Nitrogen availability is important to nitrogen cycling and plant productivity within the ecosystem. The formation of nitrogen-fixing nodules in legumes involves complex molecular interactions and recognition [61].

*Sinorhizobium* sp. and *Rhizobium* sp. are two types of soil bacteria that are capable of nodulation with alfalfa plants, including *Medicago* [62,63] and *Melilotus* spp. *Vicia* [64] and *Pisum* [65]. These strains are capable of synthesizing the distinct exopolysaccharides (EPS) succinoglycan and second exopolysaccharide (EPS II), which are both involved in symbiosis [23,24]. The precise physiological function of EPS, as produced by nodule bacteria, has been investigated in an exopolysaccharide-deficient mutant. As a result of this mutation, these bacteria failed to invade legumes and establish symbiosis due to a defect in initiating the production of infection threads [66]. Previous work has shown that exopolysaccharides are secreted in a range of sizes, represented by two major fractions: high-molecular-weight and low-molecular-weight fractions [67,68]. Moreover, the low-molecular-weight fraction represents symbiotically active forms of EPS, and it has been suggested that they may act as signaling molecules during this process [69]. During nitrogen-fixing symbiosis, lectins respond to polysaccharides produced by nodule bacteria; lectins are carbohydrate-binding proteins that promote the aggregation of rhizobia on the surface of legume root hairs, and they bind polysaccharides of the rhizobia with specific sugar-binding sites [70]. According to some reports, lectins are likely necessary within the nodule primordium to sustain cortical mitotic activity and increase the concentrations of Nod factors, which are necessary for the nodulation process [71,72].

Nod factors are lipochitooligosaccharide-based signaling molecules secreted by rhizobia that initiate nodule development in legumes. Several rhizobia genes, for example, the nodulation (*nod*) genes, are essential for successful interaction with the host, similarly to the genes involved in exopolysaccharide synthesis. Once the plant recognizes the nodulation factors, transcriptional and developmental changes occur in the root, such as cortical cell divisions, which allow bacterial invasion and nodule formation [73,74]. Due to the specificity of nodulation in hosts and the low concentrations of Nod factors, receptors for the bacterial signals are necessary. Different types of plant receptor kinases are involved in the perception of Nod factors, such as the lysine motif (LysM)-type kinase gene *NFR5* from *Lotus japonicus*, which encodes a transmembrane serine/threonine receptor-like kinase and is required for the earliest detectable plant responses to Nod factor [75], as well as the downstream component *SymRK*, which encodes a leucine-rich-repeat receptor kinase involved in nodulation symbiosis [76]. Studies have shown that individual bacterial strains can enable nodulation in a range of hosts and can synthesize a mixture of several different Nod factor molecules [72]. Furthermore, plants also choose specific rhizobium species through recognition of Nod factors [77,78]. It has been hypothesized that receptor kinases in legumes co-evolved with the structure of Nod factors to generate suitable rhizobia-root nodule symbiosis by selective perception [77].

Interestingly, the transcription of rhizobia *nod* genes is induced by root exudates. The first step toward establishing a successful symbiosis is to attract the correct plant symbiont [72]. Flavonoids released from legume roots affect root nodulation by inducing chemo-attraction of rhizobia toward the root, enhancing the growth rate of bacterial cells and inducing transcription of rhizobial *nod* genes [79]. This suggests that molecular communication within the rhizosphere is complex and interactive-flavonoids secreted by the legume root cause nodule bacteria to recognize the plant and produce Nod signaling molecules, which in turn trigger a number of processes within the root, including division of root cortical cells and nodule morphogenesis.

A recent review by Gonzalez and Marketon [16] reported that quorum sensing is involved in the signal exchange process and perhaps plays a major role in preparing and coordinating the behavior of nitrogen-fixing rhizobia during the establishment of the symbiosis. During the course of rhizobial nodulation, the bacteria undergo chemotaxis toward the plant roots, leading to an increase in cell density, and the subsequent phenomena, including nodulation, symbiosome development, exopolysaccharide production, and nitrogen fixation, all involve the QS process [16]. In *S. meliloti*, at least five different AHLs (which are produced by SinI) can induce expression of several genes involved in the biosynthesis of exopolysaccharide (EPS) and EPS-II, which play important roles during symbiosis [49]; the chemotaxis and motility of *S. meliloti* depend on the other quorum sensing regulator VisN/VisR [80]. *R. leguminosarum* has multiple QS systems (e.g., *rai*, *rhi*, *cin*, and *tra*) [16], which do not appear to play different roles during nodulation. Mutations in *cinR* and *cinI* abolish the production of *N*-(3-hydroxy-7-*cis*-tetradecenoyl)-l-homoserine lactone (3OH-C_14:1_-HSL) and also reduce the production of several other AHLs produced by *raiI*, *traI*-like, or *rhiI*. Thus, *cinIR* appears to be at the top of a regulatory cascade or network that influences several AHL-regulated QS loci, whereas mutations in *cinI* have little effect on growth or nodulation of the host plant [81].

In addition, nitrogen fixation by actinomycete-nodulated plants is a major source of biological fixation of atmospheric nitrogen [82]. The actinomycete genus *Frankia* contains nitrogen-fixing symbionts of many species of actinorhizal plants belonging to eight dicotyledonous families, which is in contrast to the rhizobium-legume symbiosis in which the host plants mainly belong to the leguminous plant family [25]. Recently, scientists have sequenced the genomes of several *Frankia* strains and uncovered no evidence of the dissemination of nodulating ability by symbiotic genes (*i.e.*, *nod* genes) in *Frankia* [83], which suggests that novel interaction mechanisms may be used during actinorhizal symbioses [84].

#### 2.1.2. Phosphate Uptake & Carbon Availability

Much of the effort devoted to studying the flavonoids exudated by roots has been focused on leguminous plants with rhizobia. However, flavonoids are also important for the establishment of mycorrhizal symbiosis, and they influence spore germination, hyphal growth and root colonization. Beneficial symbioses, such as the legume-*Rhizobium* interaction, are regulated by the production of root exudates, which stimulate the growth of fungi and influence nutrients and niches. These fungi are incapable of accomplishing their life cycle in the absence of a host root, which supplies carbon to the fungal partner [40]. Flavonoids such as glyceollin, coumestrol and daidzein have been reported to stimulate arbuscular mycorrhizal fungi (AMF) colonization in soybean [39], and it has been suggested that flavonoids act as signaling compounds during root colonization by AMF [39]. The levels of flavonoid and isoflavonoid secondary metabolites were measured in *M. truncatula* and *Medicago sativa* roots during colonization with the AMF *Glomus versiforme.* Distinct qualitative and quantitative changes in flavonoid patterns occurred during the establishment of AMF symbiosis, including transient increases in the levels of phytoalexin during the early stages of colonization [85]. It has been proposed that different concentrations and types of flavonoids in roots regulate the protective effects of AMF symbiosis against pathogens, which differ depending on the presence of beneficial or pathogenic fungi, as well as different fungal isolates or plant cultivars [22].

Strigolactones are a group of sesquiterpene lactones that induce hyphal branching in AMF, which is the critical developmental step for ensuring contact with the host root and the establishment of symbiosis [40]. It has been hypothesized that this branching factor is a plant signaling molecule that is necessary to trigger hyphal morphogenesis and root colonization during the pre-symbiotic phase [40]. Interestingly, Buee *et al.* [86] were not able to induce these effects after testing common root-exudate flavonoids and compounds synthesized via the flavonoid pathway as branching factor candidates. This finding indicates that, to some extent, complex communication must exist between plants, microorganisms, and the various bioactive compounds generated during different stages.

Although several investigations have reported that root exudates are needed for mycorrhizal fungi (MF) formation and growth [87–89], MF development is accompanied by an exchange of signaling molecules between both symbionts [27]. A diffusible fungal signaling factor that triggers gene activation in roots and is required for mycorrhization has recently been identified. This so-called “Myc factor” may be produced by AMF and recognized by host roots, and it is necessary for the establishment of successful mycorrhizal associations [27,28]. A membrane-separated co-culturing experiment provided evidence that a crucial step of fungal-host recognition requires synthesis of diffusible Myc factor [28]. This factor, produced by mycorrhizal fungi, induces elevated calcium levels and the calcium oscillations that prime epidermal root cells for fungal colonization prior to direct fungi-root contact [90]. Similarly to the symbiotic signaling pathway in legume-*Rhizobium*, it is interesting that mycorrhizal symbiosis is also controlled by the same (or similar) Myc factor receptors. Op den Camp, *et al.* [91] showed that in *Parasponia*, a single Nod factor-like receptor is indispensable for both bacterial and fungal symbiotic interactions but that legume Nod factor receptors are not essential for arbuscular endomycorrhizae.

MF can form mutualistic symbioses with the roots of approximately 80% of vascular plants, which often increase phosphate uptake and growth [52]. These associations involve the fungal mycelium uptake pathways and are beneficial to the plants, particularly during growth under nutrient-limiting conditions. A number of phosphate transporters, such as StPT3 from potato and LjPT3 from *Lotus japonicus*, show increased expression during AMF symbiosis; in contrast, OsPT11 from rice, MtPT4 from *M. truncatula*, and LePT4 from tomato, are expressed exclusively during AMF symbiosis [92]. Recently, it has been reported that the bioactive compound lyso-phosphatidylcholine (LPC), which is a signal found in mycorrhizal root extracts, is capable of inducing expression of the phosphate transporter genes *StPT3* and *StPT4* from potato, as well as *LePT4* from tomato [52]. The LPC signal might be generated preferentially by arbuscule-containing cells during AMF symbiosis [52]; however, the precise origin of this mycorrhizal signal (*i.e.*, from fungi or plants) remains unclear.

Nutrient transferring relationships between plants and AMF are a central feature of the fungal symbioses. AMF assists the host plant to acquire water and nutrients, such as phosphate and nitrogen. In return, up to 20% of the plant-fixed carbon is transferred to the fungus [27]. Hexoses formed from carbon taken up by the root were found to be the major form of carbohydrates by AMF [45]; however, triacylglycerol is the main form of stored carbon utilized by the mycobiont during all stages of its life cycle [93].

### 2.2. Survival Capacity-Virulence/Defenses

#### 2.2.1. Virulence Factors

Microorganisms within the rhizosphere are both advantageous and disadvantageous to plant growth and health. Pathogenic microorganisms, including pathogenic fungi, oomycetes, bacteria, viruses and nematodes cause many types of root diseases, such as “take-all”, rhizomania, soft rot, sudden oak death, and bacterial wilt disease, among others. These diseases are caused by secreted virulence factors that include extracellular polysaccharides, plant cell wall-degrading enzymes (CWDEs), and effector proteins (Table 1) [30,31,94,95]. Phytopathogenic fungi invade plant roots by producing enzymes to degrade cell walls, modulate turgor pressure, and colonize the root cortex [94]. The levels of certain compounds, which are taken to be pathogenicity factors, are increased in plants when pathogens begin infecting their roots. An experiment by Pasold *et al.* [47] showed that *Plasmodiophora brassicae* infection leads to strong accumulation of flavonoids in *Arabidopsis* root galls.

The fungal toxin fusaric acid (FA), which is isolated from *Fusarium heterosporum* Nees, is a potent growth inhibitor in rice seedlings, and it also proved to be toxic to plants, fungi, and bacteria; for example, it inhibited synthesis of the antimicrobial metabolite 2,4-diacetyl-phloroglucinol in *P. fluorescens* CHA0 [33]. During the infection process, strains of *Fusarium* spp. are present at the root surface, where they express several CWDEs, including endopolygalacturonase, pectatelyase, xylanase, and subtilisin-like protease; which of these specific molecules triggers pathogenicity in roots remains controversial [96]. Recent evidence suggests that two conserved signal transduction cascades—the cyclic adenosine monophosphate-protein kinase A (cAMP-PKA) and mitogen-activated protein kinase (MAPK) cascades—regulate development and virulence in *Fusarium* strains. These cascades also control plant infection in other pathogenic fungi [53].

Over the past decade, strategies to improve plant disease resistance using transgenic approaches have increased the ability of some plants to survive in soil infested with fungal pathogens. For instance, over-expression of one of the B-3 ethylene response factors (ERFs) in *Medicago* roots increased resistance to *Rhizoctonia solani* as well as *Phytophthora medicaginis*, an oomycete root pathogen [55].

#### 2.2.2. Biocontrol

Biological-control activities, operated in plants by fungi and bacteria, include competition for nutrients, the production of protective biofilms, niche exclusion, induced systemic resistance (ISR), and the production of antibiotics [1]. The increasing use of pesticides and fertilizers has several negative effects, including soil degradation and decreased resistance to environmental stresses. Therefore, the use of biocontrol agents is an attractive alternative that could reduce the amount of chemicals used in agriculture.

In many of the biocontrol systems that have been studied, one or more antibiotics have been shown to play a role in disease suppression. A wide variety of antibiotics produced by *Pseudomonas* spp., such as phenazine, pyoluteorin, 2,4-diacetyl-phloroglucinol, pyrrolnitrin, 2,3-de-epoxy-2,3-didehydro-rhizoxin, and hydrogen cyanide, can directly interfere with the growth of various pathogens and contribute to disease suppression [35]. *Bacillus subtilis*, a gram-positive biocontrol bacteria, can protect against fungal pathogen attacks by producing a variety of antibacterial agents, including a broad spectrum of lipopeptides, such as surfactin and iturin A [36]. Biofilms formed by microbes associated with roots can influence the bacteria-plant relationships and control plant disease. Bais *et al.* [97] reported that *B. subtilis* played a protective role for *Arabidopsis* roots against infection by *Pseudomonas syringae*, which was facilitated by biofilm formation. *Trichoderma* spp., *Gliocladium* spp., and actinomycetes are ecologically relevant with respect to the protection of plants against pathogens; these effects are mediated through diverse biological control mechanisms, including the production of structurally complex antibiotics such as gliovirin and gliotoxin, as well as a diverse array of bioactive compounds that inhibit the development of pathogens in the soil [98]. Recent research showed that some novel antifungal antibiotics produced by *Streptomyces* can protect plants from pathogens infection [99]. Ziedan, *et al.* [100] compared seven *Streptomyces* strains isolated from grapevine rhizospheric soil as biocontrol agents to show the antagonistic activities against *Fusarium oxysporum. Streptomyces alni* exhibited the highest antifungal activity including parasitism over *F. oxysporum* hyphae and inhibition of hyphae growth by producing antibiotics or lysis of cell.

Ectomycorrhizal symbiosis is able to alleviate toxic effects of allelopathy of plants [101]. Zeng and Mallik [101] found that *Paxillus involutus*, ectomycorrhizal fungi of black spruce, could detoxify phenolic compounds produced by *Kalmia angustifolia*. These phenolic acids and the degraded compounds were found to stimulate the growth of ectomycorrhizal fungi.

MF symbiosis can interfere with the damage caused by soil-borne plant pathogens [102]. However, this type of biocontrol of plant pathogens is usually indirect and involves nutritional improvements, alterations to root physiology [103], changes in the mycorrhizosphere microbial populations that are antagonistic to pathogens [102,104], and the activation of plant defense responses [105].

MF is commonly associated with roots in soil; however, the synergistic associations among microorganisms have also been studied with respect to their combined beneficial effects on plants [106]. Medina *et al.* [107] provided evidence showing that AMF colonization combined with the presence of *Bacillus* strains specifically changed ecological soil conditions that affected plant growth and rhizosphere microbial activity. In most cases, mycorrhizal infection increases the total number of aerobic bacteria in the rhizosphere [108], but different AMF types have different effects on bacterial and fungal populations as well as plant physiology, which can be attributed to specific changes in competition for growth substrates [107].

More obvious biocontrol effects can be observed from complex microbial interactions (involving bacteria and fungi) in the rhizosphere compared with the biocontrol effects from single agents [109–112]. A study by Roberts *et al.* [113] showed that a combination of bacterial and fungal isolates protected cucumber against damping-off, which is caused by the fungal pathogens *Rhizoctonia solani* and *Pythium ultimum*, and was also capable of suppressing the hatching of eggs from the nematode *Meloidogyne incognita in vitro*.

In most plant species, defense responses mediated by root exudates—a range of secondary metabolites and antimicrobial peptides including indole, saponins, terpenoid, benzoxazinone, flavonoid, salicylic acids, jasmonic acids, chitosans, rosemarinic acid, naphthoquinones, phytoalexins, and defensive proteins can inhibit the growth of fungal and bacterial phytopathogens [58]. Phytoalexins and 3-deoxyanthocyanidin flavonoids produced by sorghum can inhibit the growth of phytopathogenic fungi *in vitro*, establishing a novel role for flavonoids in root exudates [114]. Phenolic compounds (e.g., phenylpropanoids, indolics and flavonoids) are generated by barley (*Hordeum vulgare*) when its roots are attacked by *Fusarium graminearum* [115]. During root infection by *Fusarium*, *t*-cinnamic acid synthesis was induced at the same time, demonstrating an active and dynamic plant defense mechanism; this was the first time that *de novo* biosynthesis of root exudates had been reported in response to an attack by soil-borne pathogens. Recent studies have focused on the influence of glucosinolates and hydrolysis products on rhizosphere microbial populations [48,116]. Plant roots trigger the glucosinolate-myrosinase defense system when plant tissues are damaged, and the hydrolysis products, such as isothiocyanates, nitriles and ionic thiocyanates, could function as biocontrol agents against fungal and bacterial pathogens [48]. However, root secondary metabolites do not have consistent effects on pathogens within the rhizosphere; for example, saponinginsenosides produced by American ginseng have been identified as possessing general antifungal properties but also stimulate the growth of *Cylindrocarpon destructans*, a major soil-borne ginseng pathogen [117].

### 2.3. QS—A Messenger in Rhizosphere

Plant-growth promoting and biocontrol bacteria, such as certain *Pseudomonas* biocontrol strains, are affected by QS systems as well. Biosynthesis of antibiotics and other antifungal compounds, such as phenazines, pyrrolnitrin, 2,4-diacetylphloroglucinol, hydrogen cyanide and pyoluteorin, are related to the *phzIR* QS system in *Pseudomonas aureofaciens* [118] and the *pcoIR* system in *Pseudomonas fluorescens* [119], among others. Researchers have shown that the QS system *pcoIR* in *P. fluorescens* 2P24 controls the biocontrol activity of this agent by indirectly regulating the production of several metabolites, including 2,4-diacetylphloroglucinol, hydrogen cyanide, siderophores and proteinases that are important for its biocontrol capacity; this is in contrast to the *phzIR* QS system in *P. aereofaciens* 30–84, which is used to positively regulate the *phzFABCD* operon responsible for synthesizing phenazine [120].

Quorum sensing was also found to modulate expression of virulence genes in *P. aeruginosa*, a plant pathogen that infects the roots of *Arabidopsis* and sweet basil. Walker *et al.* [121] traced plant infection and subsequent mortality due to *P. aeruginosa* strains PAO1 and PA14 to the formation of biofilm colonies at the root surface, which were dependent on the QS system. Two AHL-mediated quorum sensing circuits have been identified in *P. aeruginosa.* The *lasIR* system has been shown to regulate expression of several virulence factors, including extracellular enzymes and toxins, and the *rhlIR* system is involved in modulating the expression of several of the virulence factors also controlled by the *las* system [50]. In contrast, the *lasIR* and *rhlIR* QS systems in the plant growth-promoting bacteria *P. aeruginosa* strain PUPa3 are involved in establishing beneficial associations with plants. These systems are important for rhizosphere colonization and act in concert to effect virulence toward *Caenorhabditis elegans* and the wax moth [122].

A study by Müller, *et al.* [123] demonstrated that AHL-mediated QS is also crucial for biocontrol activity of *Serratia plymuthica* HRO-C48, a ubiquitous inhabitant of the rhizosphere of different plant species that plays an antagonistic role to many soil-borne pathogens. The influence of AHL-mediated communication in this bacterial strain includes production of extracellular proteolytic and chitinolytic enzymes, synthesis of volatile organic compounds (VOCs) and pyrrolnitrin (which is involved in antifungal activity), and upregulation of the plant growth hormone indole-3-acetic acid.

Functional bacterial genes are expressed only when bacterial populations have reached a critical number, with either pathogenic or beneficial consequences to the host. Therefore, bacteria use quorum sensing to ensure the optimal time to activate plant responses, in order to avoid premature defense [124]. The transgenic tobacco plant was used to expression gene *expI* of *Erwinia carotovora*, the soft-rot phytopathogen, which is responsible for *N*-oxoacyl-homoserine lactone (OHL) biosynthesis. The synthesis of OHL in tobacco exhibited enhanced resistance to infection by wild-type *E. carotovora* and exogenous addition of OHL to wild-type tobacco also had a similar result [124]. The results from experiments by Toth *et al.* [125] showed that transgenic potato plants containing the gene encoding AHL synthase from *Yersinia enterocolitica* increased disease development by infection with *E. carotovora*. These results suggest that the regulation of plant cell wall-degrading enzymes by AHLs is likely a response to increased nutrient availability at later stages of infection.

Recently, this specific behavior of bacteria has also been described for fungi in the control of important processes such as biofilm formation and pathogenesis [126]. The signaling molecules, farnesol, tyrosol, dimethoxycinnamate and trisporic acid, produced by *Candida albicans*, *Uromyces phaseoli* and zygomycetes, are involved in microbe-host interactions [126]. However, there is also evidence that signals from bacteria and fungi interrelate and interact with one another. The QS signaling molecule 3-oxo-C12-HSL from *P. aeruginosa* inhibits the transition from yeast-form to filamentous growth in *C. albicans*, which is linked to virulence [51]. In turn, farnesol is able to strongly suppress AHL synthesis in *P. aeruginosa* [127]. However, the molecular pathways and the precise mechanisms of action in fungal QS systems remain unknown [126]. A study by Uroz and Heinonsalo [128] showed the potential for mycorrhizal or non-mycorrhizal root-associated fungi to degrade AHL or to prevent AHL recognition by producing quorum sensing inhibitors (QSI). This phenomenon could be a strategy developed by fungi to interfere with the deleterious bacterial functions and to control bacterial community behavior in or near plant roots.

Signaling molecules are crucial substances that coordinate the expression of certain genes and influence the activity of microbial strains within the rhizosphere. However, it is interesting that these microbial signals and sophisticated information feedback systems can be detected by and responded to by plant roots. The results from Mathesius *et al.* [129] indicate that the eukaryotic host *M. truncatula* is able to detect nanomolar to micromolar concentrations of bacterial AHLs from both symbiotic and pathogenic bacteria; the corresponding functional responses to AHLs were significantly affected, including changes in auxin balance and flavonoid synthesis proteins, as well as the secretion of plant compounds. Surprisingly, Schuhegger *et al.* [130] showed that AHLs within the rhizosphere produced by *Serratia liquefaciens* and *Pseudomonas putida* which colonized tomato roots, increased systemic resistance to the fungal leaf pathogen *Alternaria alternata* in tomato shoots. Studies in which roots were inoculated with different types of AHLs show that short chain AHLs (e.g., C4-HSL and C6-HSL) increase *Arabidopsis* root length by altering plant hormone concentrations in root and shoot tissues, while the accumulation of long chain AHLs in root tissues appears to reduce root growth [131]. Therefore, the response of plants to AHLs depends on various external factors, such as AHL type and concentration. Plants or parts of the plant will react differently to treatment with AHLs, although the mechanisms of transport and the identity of the receptor for these signaling molecules in plants are almost completely unknown.

It is possible that higher plants may also synthesize and secrete compounds that mimic the activity of bacterial AHL signaling compounds. The AHL signal-mimic activities detected in pea (*Pisum sativum*) exudates might play important roles in stimulating AHL-regulated behaviors in certain bacterial strains while inhibiting these behaviors in others [132]. This suggests that there is significant crosstalk between different bacterial species and plant roots within the rhizosphere, which is mediated through precise combinations of signal transduction and response regulation. Structures of most AHL signal-mimic compounds have not been elucidated; however, earlier findings reported that secondary metabolites from algae had structural similarities to AHL molecules [133]. Considering the inhibition of microbial growth by secondary metabolites from plants, the fact that bacterial quorum sensing systems are affected by these compounds is not surprising. l-Canavanine, an arginine analog produced by alfalfa or other legumes, inhibited AHL-signaling processes in the reporter strain *Chromobacterium violaceum* without interfering with its growth. In addition, l-cananavine appeared to regulate *S. meliloti* quorum sensing system responsible for the regulation of EPS II biosynthesis [134].

### 2.4. Other Features of Roots Exudates

Plant roots are the key source of energy or food for living organisms, so the region of soil that surrounds the root has the potential to promote the chemotaxis of soil microbes by root exudates [20]. Large quantities of organic compounds are released at the surface of roots, such as sugars, polysaccharides, amino acids, phenolic, polyacetylenes, flavonoids, fatty acids, growth regulators, nucleotides, tannins, steroids, terpenoids, alkaloids, and vitamins [20]. Researchers have tested the effects of root exudates on patterns of bacterial gene expression. Mark *et al.* [135] examined the influence of exudates from two varieties of sugar beet on the *Pseudomonas aeruginosa* transcriptome and showed that the exudates selected for genetically distinct *Pseudomonas* spp. populations within the rhizosphere. Their results showed that the majority of genes were regulated in response to only one of the two exudates. Interestingly, genes with altered expression included those with functions previously implicated in microbe-plant interactions, with effects on metabolism, chemotaxis and type III secretion. Root exudates have the potential to impact rhizosphere microbes both positively and negatively. For example, studies of chemotaxis behavior in pathogenic microbes, such as *Ralstonia solanacearum*, showed that these microorganisms depend on root exudates to locate and infect plant hosts in their natural niches [136]. The root exudates can also serve as signals between parasitic plants and host plants. These active compounds, such as haustorium inducing factors (HIFs) and 2,6-dimethoxy-1,4-benzoquinone (DMBQ), influence both germination and haustorium stage of parasitic plants [137].

In some sense, root exudates are highly plant species-specific, and they influence specific microbial communities [5]. However, these compounds secreted by plants have also shown versatility under complex below-ground conditions. For example, the nodulation genes of nitrogen-fixing bacteria are induced by flavones and isoflavones, which are beneficial for leguminous plants, while zoospores of *Phytophthora sojae*, a soybean pathogen, are specifically attracted to isoflavones for host recognition and infection initiation [138]. Flavonoids have various functions within the rhizosphere with respect to the interaction of roots with microorganisms, including chemoattraction, stimulating rhizobial *nod* gene expression, mycorrhizal spore germination, and inhibiting root pathogens (as mentioned above), as well as chelating soil nutrients, affecting quorum sensing, and mediating allelopathic interactions between plants [56,139,140].

Recent review summarized some flavonoids involved in allelopathic inhibitor of seedling growth, such as 5,7,4′-trihydroxy-3′,5′-dimethoxyflavone, quercetin and kaempferol [140]. Plant roots secrete allelochemicals as phytotoxins, which mainly exert their influence through resource competition and inhibition of germination and seedling growth in neighboring plants. These detrimental interactions are also described as plant defenses in response to stress or local rhizosphere conditions [141]. Allelochemicals from bigalta limpograss (*Hemarthria altissima*) root are mainly phenolic compounds that serve as plant growth inhibitors [142]. These phenolic compounds were analyzed by gas chromatography-mass spectrometry and were found to contain 3-hydroxyhydrocinnamic, benzoic, phenylacetic, and hydrocinnamic acids, which are major rhizospheric compounds with known growth-regulatory activities. The effects of root exudates on ion uptake by cucumber seedlings were examined using phenolic acids, such as cinnamic acid, vanillic acid, and ferulic acid. Among the compounds tested, *o*-hydroxybenzoic acid showed the strongest effect on nutrient absorption in cucumber [143].

## 3. Conclusions and Perspectives

The metabolism of biological communities in and near roots within the soil promotes energy cycling at microscopic scales, and the scientific studies of these unseen below-ground ecosystems are growing rapidly. The development of below-ground systems is accompanied by an exchange of bioactive molecules between roots and microbes. Significant effort has been expended to elucidate the complex communication systems used by plants and microorganisms and to identify the various bioactive compounds generated by different species. Generally, the type and structure of bio-molecule can analyzed by gas chromatography-mass spectrometry, high-performance liquid chromatography-mass spectrometry, nuclear magnetic resonance and some biological detection methods [1,12,40]. By taking advantage of specific biological chemicals to effect growth stimulation or suppression, future studies will be able to test the contribution of these compounds with respect to terrestrial ecosystem biodiversity, variability and productivity.

**Figure 2:**
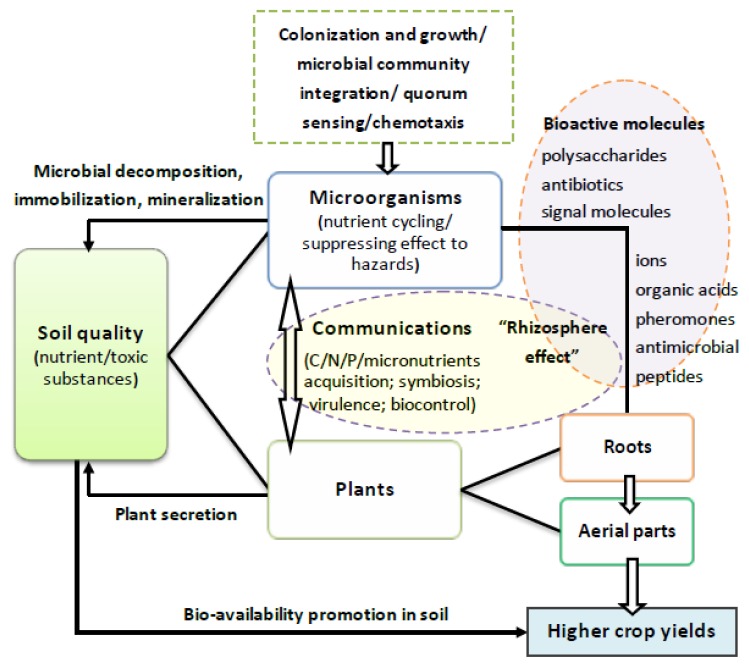
Net structure of rhizospheric interactions between microorganisms and plants playing critical roles in below-ground ecosystem and sustainability of agriculture. [Contribution of rhizosphere effect generated by microbes and roots appears in the improvement of soil nutrients acquisition and bioabsorbable and biotransformation efficiency. Bioactive molecules used by roots and microbes for communication can influence: (1) microbial behavior (*i.e.*, chemotaxis, colonization, growth, and group behavior); and (2) root growth and crop productivity.]

The development of agriculture has been accompanied by the increasing use of fertilizers, water, pesticides, new crop strains, and other technologies to increase production [144]. However, soil degradation and productivity loss are a byproduct of these unsustainable agricultural practices. In addition, the rate of increase in crop production using traditional physical and chemical control methods has appeared to stall [3,145,146]. Therefore, recent interest has focused on possible strategies to ensure the sustainability of agriculture using multifactorial responses in the rhizosphere [147]. The potential of microbes to accelerate below-ground circulation of nutrients may provide the key to increasing plant yields. Moreover, suppressing the effects of harmful substances supplied by microorganisms can also reduce damage to plants. Therefore, a full understanding of rhizospheric processes and soil-microbe-plant ecosystems will require the analysis of many variables, such as soil conditions, functional microbial species evolution, community integration, unique bioactive molecules used for survival, regulation of growth and communication, metabolic regulation in plants, and the coordination of mechanisms in aerial plant parts and roots. Together, these will provide the knowledge to develop new systems for controlling the stability of complex ecosystems both above and below-ground (Figure 2).

Based on the significant interactions between microbes and plant roots, practices that utilize effects within the rhizosphere to improve yields will be crucial. For example, one way to improve biocontrol within the rhizosphere may be to add mixtures or combinations of biocontrol agents, particularly if they exhibit different or complementary modes of action or abilities to colonize root microsites [111]. For another instance, the development of genetic engineering methods within the rhizosphere has provided opportunities to encourage beneficial microbes or to select against pathogens in transgenic plants, to modify plant-growth promoting rhizobacteria (PGPR) to release antibiotics for pathogen suppression and enhance nutrient acquisition, and to interfere with QS signals essential to pathogen life cycles [148,149]. Although some biotechnological methods meant to influence yields by altering features of the rhizosphere environment have been tested, practical applications in this field remain difficult to implement due to the large number of variables to consider, including the identification of key biological species, and the complex effects between biological processes and chemical molecules. Relatively little is known regarding signaling responses and regulatory mechanisms within complex below-ground environments. For example, within the rhizosphere, the spatial distribution of cells might be more important for quorum sensing than cell density [150]. Furthermore, the signals emitted from microorganisms and plant cells change in space and time as roots and microbes pass through different stages of their life cycle, diversifying the release and combinations of molecules, which makes studying rhizosphere communities very complex [72]. Therefore, new strategies for optimizing soil-ecology and increasing yield will only receive increasing attention, and further research to explore the viability of these strategies in practice will be needed.

## Figures and Tables

**Table 1:** Some typical primary bioactive molecules showed direct effects in rhizosphere. (Primary rhizosphere effects contain beneficial or adverse influences (e.g., symbiosis, biocontrol, and pathogenicity) on plant or microbial growth and survival. The major bio-molecules present in below-ground ecosystems include microbial products and root exudates, respectively.)

Primary rhizosphere effects	Bio-molecules	Agents involved	Functional description/Recipients	References
**Microorganisms driving**				
Nitrogen fixation	Exopolysaccharides: EPS II; succinoglycan	*Sinorhizobium meliloti; Rhizobium* sp.	Nodulation with a majority of leguminous plants (*Medicago* and *Melilotus* spp.; *Vicia; Pisum; Parasponia*) and other plants	[23–25]
	Nodulation factors: lipochitooligosaccharide	*Rhizobium meliloti*	Inducing a variety of effects including deformation of root hairs, division of root cortical cells, and nodule morphogenesis	[26]
Symbionts (with Arbuscular mycorrhizal fungi)	“Myc factor” (soluble signaling molecules)	Arbuscular mycorrhizal fungi	Fungal signaling factor that triggers gene activation in the root required for mycorrhization	[27,28]
Metal uptake	Glutathione; metallothioneins	Ectomycorrhizal fungi	Influence on metallic element bioavailability in soil	[29]
Virulence factors	Extracellular polysaccharide	*Pseudomonas solanacearum*	Responsible for the wilt symptoms	[30]
	Extracellular plant cell wall-degrading enzymes	*Ralstonia solanacearum*		[31]
	Effector proteins	*Pseudomonas syringae; Xanthomonas spp.; Ralstonia solanacearum; Erwinia species*	Essential for the virulence and suppression of host defense responses	[32]
	Phytotoxin (fusaric acid)	*Fusarium oxysporum*	Inhibiting the growth of rice seedlings and repressing antimicrobial activity of the biocontrol strain *Pseudomonas fluorescens* CHA0	[33]
	*m*-hydroxyphenylacetic acid; *m*-methoxyphenylacetic acid	*Rhizoctonia solani*	Infection of soybeans and decreasing of nodule formation	[34]
Biological control activities	Antibiotics: phenazine; pyoluteorin; 2,4-diacetyl-phloroglucinol; pyrrolnitrin; 2,3-de-epoxy-2,3-didehydro-rhizoxin; hydrogen cyanide	*Pseudomonas* spp.	Interfering growth of various pathogens and contributing to disease suppression	[35]
	Lipopeptides: surfactin; iturin A	*Bacillus subtilis*	Antibacterial and antifungal agents	[36]
	Antibiotics: gliovirin; gliotoxin	*Trichoderma* spp.; *Gliocladium* spp.	Protection of plants against pathogens	[37]
**Roots driving**				
Bacterial symbionts	Flavonoids	*Medicago truncatula*	Stimulating presymbiotic steps in rhizobia	[38]
Fungal symbionts (with Arbuscular mycorrhizal fungi)	Flavonoids: glyceollin; coumestrol; daidzein	*Glycine max*	Root colonization by mycorrhizal fungi	[39]
	Strigolactone	*Lotus japonicas*;	Branching factor (hyphal branching of AMF) that precedes successful root colonization	[40]
	Jasmonic acid	*Hordeum vulgare* cv Salome	Colonization rate and arbuscule formation in mycorrhizal roots	[41]
	Auxin and auxin conjugates	*Zea mays*	Enhanced fungal growth	[42]
	Gibberellin	*Nicotiana tabacum*	Strengthening the carbohydrate sink of the fungi	[43]
	Abscisic acid; ethylene	*Lycopersicon esculentum*	Development of the complete arbuscule and its functionality	[44]
Carbon availability	Hexose	*Medicago truncatula; Daucus carota*	Carbon uptake and metabolism	[45,46]
Pathogenicity factors and defence response	Flavonoids	*Arabidopsis*	An intense accumulation of flavonoids in *Arabidopsis* root infected by *Plasmodiophora brassicae*	[47]
	Phytoalexins: indole; saponins; terpenoid; benzoxazinone; flavonoid; rosmarinic acid; naphthoquinones,	--	Defence compounds of the rhizosphere against pathogenic microorganisms	[1]
	Glucosinolates and hydrolysis products (isothiocyanates; nitriles; ionic thiocyanates)	*Arabidopsis thaliana*	Against fungal and bacterial pathogens	[48]

--: means it’s not a specific description.

**Table 2:** Secondary bioactive molecules of indispensable regulatory mechanisms in rhizosphere. (Indirect effects contain various cell-signaling networks to control biological activities during plant-microbe communication processes. The major bio-molecules served as signals in below-ground ecosystems include microbial products and root exudates, respectively.)

Indirect effects	Bioactive compounds	Agents involved	Functional description/Recipients	References
**Microorganisms driving**				
Quorum sensing	*N*-acyl homoserine lactones (AHLs); *p*-coumarate; quinolone	Gram-negative bacteria	Cell-cell communication between bacteria to regulate symbiotism, virulence, swarming behavior, biofilm formation and antibiotic production	[11,12,14–17,49,50]
	Oligopeptides	Gram-positive bacteria		
	AI-2: furanosyl borate diester	--		
Fungal QS systems	Farnesol; tyrosol; dimethoxycinnamate; trisporic acid	*Candida albicans; Uromyces phaseoli*; zygomycetes	Controlling biofilm formation and pathogenesis in fungus	[51]
Phosphate acquisition (with Arbuscular mycorrhizal fungi)	Lysophosphatidylcholine	Arbuscular mycorrhizal fungi; *Solanum tuberosum* L.; *Solanum lycopersicum* L. *	Induction of plant phosphate transporter gene and mycorrhiza formation	[52]
Virulence	Signal transduction cascades: cAMP-PKA and MAPK cascade	*Fusarium* strains	Sensing environmental cues and respond by appropriate changes in gene expression to establish disease	[53]
**Roots driving**				
Defence response	NAD(P)H oxidases, phospholipases, phosphatases and protein kinases; linolenic acid; jasmonic acid; methyl jasmonate	--	The low doses might act as signals for activation of other defence reactions	[54]
	B-3 ethylene response factors (ERFs)	*Medicago*	Resistance to *Rhizoctonia solani* and *Phytophthora medicaginis*	[55]
Complex effects	Flavonoids	--	Stimulating or inhibiting rhizobial *nod* gene expression, causing chemoattraction of rhizobia towards the root, inhibiting root pathogens, stimulating mycorrhizal spore germination and hyphal branching, mediating allelopathic interactions between plants, affecting quorum sensing, and chelating soil nutrients	[56]

-- means it’s not a specific description;

* means the origin of this signal is still unsure.
